# Learning Curves of Macintosh Laryngoscope in Nurse Anesthetist Trainees Using Cumulative Sum Method

**DOI:** 10.1155/2014/850731

**Published:** 2014-02-12

**Authors:** Panthila Rujirojindakul, Edward McNeil, Rongrong Rueangchira-urai, Niranuch Siripunt

**Affiliations:** ^1^Department of Anesthesiology, Faculty of Medicine, Prince of Songkla University, Hat Yai, Songkhla 90110, Thailand; ^2^Epidemiology Unit, Faculty of Medicine, Prince of Songkla University, Hat Yai, Songkhla 90110, Thailand

## Abstract

*Background*. Tracheal intubation is a potentially life-saving procedure. This skill is taught to many anesthetic healthcare professionals, including nurse anesthetists. Our goal was to evaluate the learning ability of nurse anesthetist trainees in their performance of orotracheal intubation with the Macintosh laryngoscope. *Methods*. Eleven nurse anesthetist trainees were enrolled in the study during the first three months of their training. All trainees attended formal lectures and practice sessions with manikins at least one time on performing successful tracheal intubation under supervision of anesthesiology staff. Learning curves for each nurse anesthetist trainee were constructed with the standard cumulative summation (cusum) methods. *Results*. Tracheal intubation was attempted on 388 patients. Three hundred and six patients (78.9%) were successfully intubated on the trainees' first attempt and 17 patients (4.4%) on the second attempt. The mean ± SD number of orotracheal intubations per trainee was 35.5 ± 5.1 (range 30–47). Ten (90.9%) of 11 trainees crossed the 20% acceptable failure rate line. A median of 22 procedures was required to achieve an 80% orotracheal intubations success rate. *Conclusion*. At least 22 procedures were required to reach an 80% success rate for orotracheal intubation using Macintosh laryngoscope in nonexperienced nurse anesthetist trainees.

## 1. Introduction

Tracheal intubation is a life-saving procedure that is mostly performed by a doctor, such as an anesthesiologist. A failed intubation or a difficult intubation is an important cause of morbidity and mortality associated with direct airway trauma and hypoxia [1, 2]. Nowadays, various novel video laryngoscopes have been developed for intubation [1–7], but these instruments are expensive and are not available in community hospitals where the Macintosh laryngoscope, a conventional laryngoscope, is still widely used.

Mastery of intubation with a Macintosh laryngoscope is necessary in clinical practice for all medical personnel, not just the anesthesiologist. The nurse anesthetist not only takes care of the patient during anesthesia but also manages the patient's airway. In Thailand, there are an inadequate number of anesthesiologists. Nurse anesthetists are therefore often required to intubate the patients during anesthesia. Their level of experience in performing this difficult procedure successfully, thus, needs to be determined.

The cumulative summation (cusum) technique was originally introduced to monitor outcomes in industrial quality control [8–10]. More recently, cusum charts are used to assess trends and proficiencies of surgical procedures [11–13], anesthetic procedures [14–17], and airway equipment use [1–7, 18, 19]. The cusum method determines if a procedure is acceptable or unacceptable using a binary outcome, such as a successful or failed intubation. This quantitative measure of performance can be used to evaluate trainee's performances and help to improve training programs.

The purpose of this study was to determine the learning ability of nurse anesthetist trainees based on the number of attempts required before a successful orotracheal intubation with Macintosh laryngoscope using the cusum method.

## 2. Materials and Methods

Following the Institutional Ethics Committee approval, this longitudinal study was conducted in a tertiary care university hospital. Twelve nurse anesthetist trainees gave written informed consent to participate in the study during the first three months of their training, between October 10, 2011 and January 15, 2012. All trainees completed a questionnaire about their endotracheal intubation experiences. One trainee was excluded because she intubated more than 40 cases, and her level of experience was considered too high. The principal investigator created the logbook and gave it to each nurse anesthetist trainee to record all intubated data immediately after each procedure.

Before the study commenced, all trainees were given a formal lecture about endotracheal intubation. Each trainee practiced with a manikin under an instructor's supervision until a successful orotracheal intubation using Macintosh laryngoscope was performed. Each laryngoscopy was done under supervision of the attending anesthesiologist staff. For this reason, the Institutional Ethics Committee waived the requirement for patients to sign a consent form.

The anesthesiologist staff assigned the elective surgical patients to the trainees for premedication and intubation. Patients with anticipated difficult intubation or cardiothoracic or airway surgery were not assigned to the nurse anesthetist trainee. The trainees recorded the airway assessment, including thyromental distance, interincisor gap, modified Mallampati classification, upper lip bite test classification, and neck movement, in their logbooks. The attending staff anesthesiologist selected the size of endotracheal tube and the Macintosh laryngoscope blade. Before intubation, the patient's head position was in the “sniffing” position. Muscle relaxant was administered before the orotracheal intubation in all patients. External laryngeal manipulation or the backward upward rightward pressure (BURP) maneuver was allowed to be performed as appropriate. Mask ventilation was allowed during each intubation attempt. The trainee recorded intubated data including laryngoscopic view by using Cormack-Lehane (CL) classification, duration of intubation, number of intubation attempts, difficulty of intubation, and confidence in performing a successful intubation. Patients who had nasotracheal intubation and/or received a video laryngoscope were not recorded in the logbook. Serious complication from prolonged intubation time, oxygen desaturation, was recorded.

After three months, the trainees sent their complete logbooks to the principal investigator. Data were collected from their logbooks and entered into a computer.

### 2.1. Variables

Duration of intubation was defined as the time from insertion of the blade between the teeth until the endotracheal tube was placed in the trachea. If the laryngoscopy was attempted more than once, the duration of intubation was recorded for the successful intubation. The trainees were allowed up to 3 attempts to intubate the patients before their supervisor took over. A successful intubation was confirmed by the capnography. A failed intubation was recorded if the endotracheal tube was not placed in the trachea. Difficulty of intubation was divided into 5 grades, very easy, easy, moderate, difficult, and very difficult, and was self-assessed. Confidence of successful intubation was self-assessed by the trainee and ranged from 0–100%. Oxygen desaturation was defined as pulse oximeter less than 95%.

### 2.2. Cumulative Summation (Cusum) and Risk Adjusted Calculation

Cusum was calculated by using the formulas *a* = ln⁡((1 − *β*)/*α*), *b* = ln⁡((1 − *α*)/*β*), *P* = ln⁡(*p*_1_/*p*_0_), *Q* = ln⁡((1 − *p*_0_)/(1 − *p*_1_)), *S* = *Q*/(*P* + *Q*), *h*_0_ = *b*/(*P* + *Q*), *h*_1_ = *a*/(*P* + *Q*), *n* = (*h*_0_∗(1−*α*) − *α*∗*h*_1_)/(*S* − *p*_0_), and *m* = (*h*_1_∗(1 − *β*) − *β*∗*h*_0_)/(*p*_1_ − *S*). Symbols used in the formula were as follows: *p*_0_ was defined as the acceptable failure rate, *p*_1_ was defined as the unacceptable failure rate, *α* was defined as the probability of wrongly accusing a trainee of unacceptable performance or type 1 failure rate, *β* was defined as the probability of wrongly certifying a trainee's performance to be acceptable or type 2 failure rate, *n* was defined as the expected number of attempts to cross *h*_0_ under given failure rate *p*_0_, and *m* was defined as the average number of attempts to cross *h*_1_ under given failure rate *p*_1_.

Risk-adjusted cusum was calculated from the observed minus expected (*O* − *E*) cusum method. A risk score of each patient was calculated as the estimated probability of failure predicted as the risk factors of the difficult intubation by using logistic regression. The risk-adjusted cusum chart was calculated by adding 1 minus the individual patient risk score to the cumulative score for each failure and subtracting the risk score for each failed attempt. The cusum at time *t*  (*c*_*t*_) is, then, *c*_*t*_ = *c*_*t*−1_ + (*x*_*t*_ − *x*_0_), where *c*_*t*−1_ is the cusum through the previous attempt, *x*_*t*_ is 1 for failure and is 0 for success (observed), and *x*_0_ is the estimated risk for the patient being intubated.

### 2.3. Sample Size Calculation

The sample size for minimal practice procedure was calculated based on using cusum calculation, an acceptable failure rate (*p*_0_) of 20%, and an unacceptable failure rate (*p*_1_) of 40%. The type I (*α*) and type II (*β*) errors were set to 0.1. The expected number of attempted procedures to cross the lower decision limit (*h*_0_) and average number of attempted procedures to cross the upper decision limit (*h*_1_) were 19 and 17, respectively. We assumed that the nurse anesthetist trainees would each practice between 25 and 30 procedures of orotracheal intubation during the first three months of training.

### 2.4. Statistical Analysis

For categorical variables, proportions were compared using Chi-squared or Fisher's exact test. Continuous variables were analyzed using Student's *t*-test or Wilcoxon's rank sum test. Multivariate analysis was performed by a fitting a logistic regression model including all variables having a *P* value <0.1 from the univariate analysis. A stepwise backward elimination procedure was used to obtain the final model predicting a successful intubation. The final model was determined by selecting the model that had the lowest value of Akaike's information criterion (AIC) at each step. Standard cusum and risk-adjusted cusum techniques were calculated by using the formula shown as above. Both standard and risk-adjusted cusum charts were calculated for each trainee. A limitation of the standard cusum method is that it does not allow weighting of the cusum score based on the expected difficulty of each procedure. From the risk-adjusted *O* − *E* cusum analysis, trainees whose performance reflects the average across trainees for similarly difficult intubations will have values close to the zero line. The median and 95% confidence interval of overall number of attempts to cross *h*_0_ with 20% acceptable failure rate was calculated by using the Kaplan-Meier method. A *P* value <0.05 was considered statistically significant. All analyses were performed using R version 2.15.3.

## 3. Results

Eleven female nurse anesthetist trainees participated in this study. Of these, only one had previous experiences of intubation, but was successful in only one of these. The other 10 trainees had no intubation experience. The mean (standard deviation; SD) age of all trainees was 24 (1.6) years. Four hundred and twelve laryngoscopies were performed on 388 patients, of which 323 laryngoscopies (78.4%) were successful, 306 (94.7%) on the first attempt and 17 (5.3%) on the second attempt. Eighty-nine laryngoscopies (21.6%) in 65 patients were failures, 82 (92.1%) on the first attempted laryngoscopy, six (6.8%) on the second attempt, and one (1.1%) on the third attempt. The median (interquartile range (IQR)) duration of intubation was significantly lower in the successful intubations (50 seconds; IQR = 35−75 seconds) than in the failed intubations (65 seconds; IQR = 50−90 seconds, *P* < 0.001). All patients' characteristics are shown in Table 1. Age, interincisor gap, Mallampati classification, and laryngoscopic view were significantly different between the success and failure groups. After logistic regression analysis, Mallampati classification, interincisor gap, and laryngoscopic view were significant independent patient factors associated with a successful intubation (Table 2) and the model containing these three variables was used to calculate the risk scores for successful orotracheal intubation.

**Figure 1:**
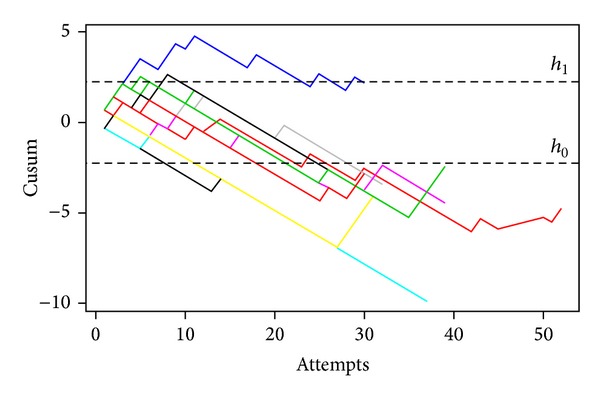
Cumulative sum chart for orotracheal intubation. Each color line and each number represent the learning curves of each nurse anesthetist trainee. Lines *h*_0_ and *h*_1_ represent the upper and lower decision limit of 2.24 and −2.24, respectively.

**Figure 2:**
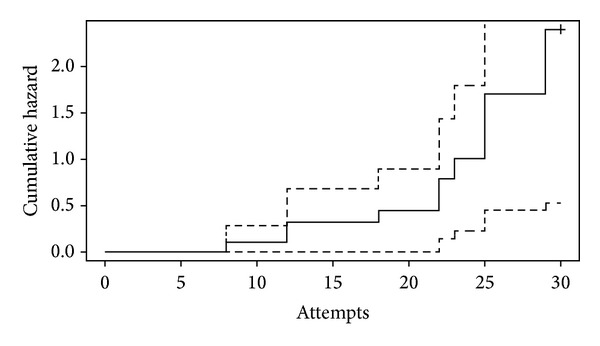
Orotracheal intubation cumulative hazard curve for number of attempts to cross *h*_0_ with 20% acceptable failure rate.

**Figure 3:**
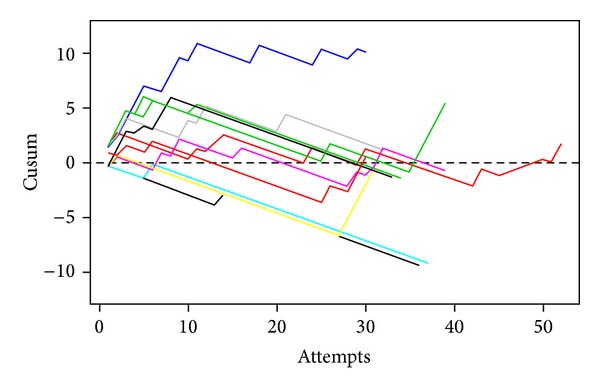
Risk-adjusted cusum chart for orotracheal intubation. Each color line represents the learning curves of each nurse anesthetist trainee.

The mean ± SD number of intubation attempts per trainee was 35.5 ± 5.1 (range 30–47). Ten (90.5%) trainees crossed the 20% acceptable failure rate line from the cusum analysis (Figure 1).  Table 3 shows the number of attempts needed to cross a 20% acceptable failure rate. From Kaplan-Meier method, a median of 22 (95% confidence interval (CI) = 18–Infinity) attempts was required to achieve an 80% orotracheal intubation success rate (Figure 2). Nine (82%) trainees crossed the zero line by using the risk-adjusted *O* − *E* cusum (Figure 3). Oxygen desaturation and dental trauma were not found in any patient.

For self-assessment, the median (IQR) level of confidence in performing a successful intubation was significantly higher when the trainees could successfully intubate their patients (median = 70%, IQR = 60%−80% versus median = 50%, IQR = 30%−50%).

## 4. Discussion 

Using the cusum method, our study found that 22 procedures were required to achieve an 80% orotracheal intubation success rate using Macintosh laryngoscope by nurse anesthetist trainees. Our result was similar to the study of Komatsu et al. [9] which reported that 29 procedures were required for a successful intubation in nonexperienced interns. The criteria for successful tracheal intubation (allowing three laryngoscopy attempts and providing external laryngeal pressure) were similar in both studies. Fewer procedures were required by our trainees compared to Komatsu et al. [9] for two reasons. Firstly, anticipated difficult airway cases were excluded from our study because these cases were excluded by the more experienced anesthesiologists. Secondly, the BURP maneuver, which helps to improve visualization of the larynx and thus facilitate intubation, was allowed to be performed as appropriate depending on attending staff anesthesiologist recommendation. Other studies [14, 20, 21] found that a higher number of procedures (43–75 attempts) were required for achieving successful intubation than in our study, probably because these studies had a stricter criteria for successful intubation (only one laryngoscopy allowed without physical assistance) and included all types of patients, including those with difficult intubations.

Our study found that 82% of trainees crossed the zero line using the risk-adjusted *O* − *E* cusum, a result that was higher than that reported by Komatsu et al. [9] (60% of interns). This may be because our study included fewer variables for predicting successful intubation.

The serious complication from prolonged duration of intubation, oxygen desaturation, was not found in this study because we allowed ventilation during each attempted intubation, and rapid sequence induction cases were excluded.

The level of confidence in performing a successful intubation was significantly higher when the trainees intubated successfully. However, this was self-assessed, the results may be biased.

Our results were used to quantitatively monitor the competency of individual nurse anesthetist trainees for orotracheal intubation with Macintosh laryngoscope. We explored the intubated cases of the trainees who did not achieve successful intubation to see whether or not their cases were different from those of other trainees who achieved successful intubation. Then, we gave feedback and notified other anesthesiologist staff to closely monitor those trainees who failed intubation. Finally, we assigned more cases to the trainees who failed intubation for more practice so that their skills could be improved.

The limitations of this study were that it was conducted in only one teaching institution and the number of maneuvers used to obtain a successful intubation was not recorded. The results may not be generalizable to other teaching institutions because staff training techniques may differ.

## 5. Conclusion

Nonexperienced nurse anesthetist trainees require 22 procedures to achieve an 80% orotracheal intubation success rate with Macintosh laryngoscope.

## Figures and Tables

**Table 1:** Patient characteristics and airway assessment details.

	Successful intubation (*n* = 323)	Failed intubation (*n* = 65)	*P* value
Age (yrs)	41 (29–53)	47 (32–60)	0.05
BMI (kg/m^2^)	22.5 (20.2–25.2)	22.8 (19.7–25.3)	0.99
ASA physical status			0.87
1	71 (22)	13 (20.3)	
2	244 (75.8)	49 (76.6)	
3	7 (2.2)	2 (3.1)	
Thyromental distance (FB)			0.42
≤3	155 (48.0)	27 (41.5)	
3	168 (52.0)	37 (58.5)	
Limit flexion and extension of neck	3 (0.9)	2 (3.1)	0.20
Interincisor gap (FB)			0.03
2	7 (2.2)	4 (6.3)	
3	291 (90.1)	60 (92.3)	
4	25 (7.7)	1 (1.5)	
Upper lip bite test classification			0.71
1, 2	312 (96.6)	62 (95.4)	
3	11 (3.4)	3 (4.6)	
Mallampati classification			0.002
1, 2	315 (97.5)	57 (87.3)	
3, 4	8 (2.5)	8 (12.7)	
Laryngoscopic view			<0.001
1, 2	320 (99.1)	51 (78.5)	
3, 4	3 (0.9)	14 (21.5)	

Data are median (interquartile range) or number (percentage).

BMI: body mass index; ASA: American Society of Anesthesiologists; FB: fingerbreadth.

**Table 2:** Significant patient factors for successful intubation.

Factor	Crude OR (95% CI)	Adjusted OR (95% CI)	*P* value (Wald's test)	*P* value (LR test)
Interincisor gap (Ref. = 2)				0.11
3	2.77 (0.79–9.77)	2.41 (0.59–9.92)	0.22	
4	14.29 (1.37–149.1)	10.2 (0.89–116.5)	0.06	
Mallampati class (Ref. = 1, 2)				0.02
3, 4	0.18 (0.07–0.5)	0.23 (0.07–0.72)	0.01	
Laryngoscopic view grade (Ref. = 1, 2)				<0.001
3, 4	0.03 (0.01–0.12)	0.04 (0.01–0.14)	<0.001	

OR: odds ratio; CI: confidence interval; Ref.: reference group; LR: likelihood ratio.

**Table 3:** Individual results.

Nurse anesthetist trainee	Attempts	Successes	Success rate (95% CI)	Number of attempts needed to cross *h*_0_
1	36	35	0.97 (0.85, 0.99)	8
2	47	38	0.81 (0.67, 0.91)	23
3	34	29	0.85 (0.69, 0.95)	25
4	30	19	0.63 (0.44, 0.80)	Not crossed
5	37	36	0.97 (0.86, 0.99)	12
6	39	32	0.82 (0.86, 0.92)	22
7	31	26	0.83 (0.66, 0.95)	12
8	32	26	0.81 (0.64, 0.93)	29
9	33	28	0.85 (0.68, 0.95)	25
10	30	24	0.80 (0.61, 0.92)	18
11	39	30	0.77 (0.61, 0.89)	22

Total	388	323	0.83 (0.79, 0.87)	Median = 22

CI: confidence interval.
